# (2*SR*,3*RS*)-Benz­yl[4-chloro-1-(4-chloro­phen­yl)-1-methoxy­carbon­yl-2-but­yl]­ammonium chloride

**DOI:** 10.1107/S1600536808030742

**Published:** 2008-09-27

**Authors:** Åsmund Kaupang, Marianne Bolsønes, Thywill Gamadeku, Tore Hansen, Martin Johanson Hennum, Carl Henrik Görbitz

**Affiliations:** a Department of Chemistry, University of Oslo, PO Box 1033 Blindern, N-0315 Oslo, Norway

## Abstract

In the racemic hydro­chloride salt of the title ester, C_19_H_22_Cl_2_NO_2_^+^·Cl^−^, the penta­noic acid chain shows a mixture of *trans* and *gauche* orientations to give an overall helical conformation. The dihedral angle between the two aromatic rings is 26.11 (10)°. The charged secondary amine function participates in two N—H⋯Cl hydrogen bonds.

## Related literature

For a related structure, see: Froimowitz *et al.* (1998 ▶).
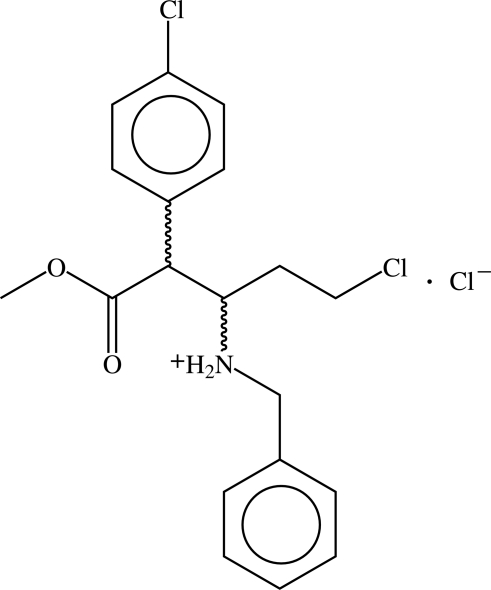


## Experimental

### 

#### Crystal data

C_19_H_22_Cl_2_NO_2_^+^·Cl^−^
*M* *_r_* = 402.73
Triclinic, 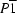

*a* = 9.263 (2) Å
*b* = 10.432 (3) Å
*c* = 11.490 (3) Å
α = 115.954 (3)°
β = 93.925 (3)°
γ = 103.015 (3)°
*V* = 954.8 (4) Å^3^
*Z* = 2
Mo *K*α radiation
μ = 0.49 mm^−1^
*T* = 296 (2) K
0.60 × 0.38 × 0.18 mm

#### Data collection

Bruker APEX II CCD diffractometer
Absorption correction: multi-scan (*SADABS*; Sheldrick, 1996 ▶) *T*_min_ = 0.782, *T*_max_ = 0.915
8767 measured reflections
4422 independent reflections
3320 reflections with *I* > 2σ(*I*)
*R*_int_ = 0.024

#### Refinement

*R*[*F* ^2^ > 2σ(*F* ^2^)] = 0.046
*wR*(*F* ^2^) = 0.159
*S* = 1.03
4422 reflections
227 parameters
H-atom parameters constrained
Δρ_max_ = 0.43 e Å^−3^
Δρ_min_ = −0.36 e Å^−3^

### 

Data collection: *APEX2* (Bruker, 2007 ▶); cell refinement: *SAINT-Plus* (Bruker, 2007 ▶); data reduction: *SAINT-Plus*; program(s) used to solve structure: *SHELXTL* (Sheldrick, 2008 ▶); program(s) used to refine structure: *SHELXTL*; molecular graphics: *SHELXTL*; software used to prepare material for publication: *SHELXTL*.

## Supplementary Material

Crystal structure: contains datablocks I, global. DOI: 10.1107/S1600536808030742/hb2801sup1.cif

Structure factors: contains datablocks I. DOI: 10.1107/S1600536808030742/hb2801Isup2.hkl

Additional supplementary materials:  crystallographic information; 3D view; checkCIF report

## Figures and Tables

**Table 1:** Hydrogen-bond geometry (Å, °)

*D*—H⋯*A*	*D*—H	H⋯*A*	*D*⋯*A*	*D*—H⋯*A*
N1—H2⋯Cl3	0.90	2.28	3.0890 (18)	150
N1—H1⋯Cl3^i^	0.90	2.59	3.2447 (18)	131

Symmetry code: (i) 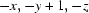.

**Fig. 1.**
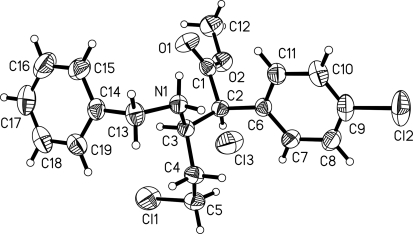
: The molecular structure of (I). Displacement ellipsoids are shown at the 50% probability level and H-atoms are shown as spheres of arbitrary size.

**Fig. 2.**
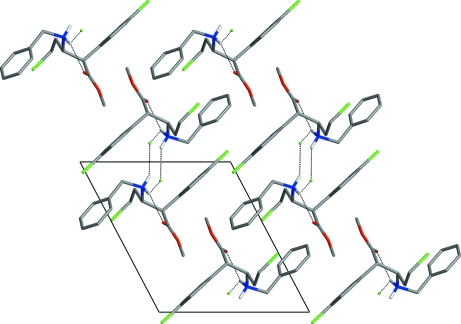
: Molecular packing and unit cell of (I) viewed along the a axis. Hydrogen bonding is indicated by dashed lines, H-atoms bonded to C have been omitted for clarity.

## supplementary crystallographic information

### Comment

The title molecule, (I), is a β-amino acid ester resulting from a rhodium-catalyzed C—H insertion reaction between methyl-(4-chlorophenyl)diazo acetate and Boc-protected *N*-benzyl-3-chloropropanamine. Full details will be published elsewhere.

### Experimental

Colourless blocks of (I) were grown by diffusion of diethylether into 100 µl of a solution containing about 5 mg of the title compound in methanol.

### Refinement

The H atoms were positioned with idealized geometry (N–H = 0.90 Å, C–H = 0.93-0.98 Å) and refined as riding with *U*_iso_(H) = 1.2*U*_eq_(C, N) or 1.5*U*_eq_(methyl C).

### Crystal data

**Table:**

C_19_H_22_Cl_2_NO_2_^+^·Cl^−^	*Z* = 2
*M**_r_* = 402.73	*F*(000) = 420
Triclinic, *P*1	*D*_x_ = 1.401 Mg m^−^^3^
Hall symbol: -P 1	Mo *K*α radiation, λ = 0.71073 Å
*a* = 9.263 (2) Å	Cell parameters from 3509 reflections
*b* = 10.432 (3) Å	θ = 2.0–28.7°
*c* = 11.490 (3) Å	µ = 0.49 mm^−^^1^
α = 115.954 (3)°	*T* = 296 K
β = 93.925 (3)°	Block, colourless
γ = 103.015 (3)°	0.60 × 0.38 × 0.18 mm
*V* = 954.8 (4) Å^3^	

### Data collection

**Table:**

Bruker APEXII CCD diffractometer	4422 independent reflections
Radiation source: fine-focus sealed tube	3320 reflections with *I* > 2σ(*I*)
graphite	*R*_int_ = 0.024
Detector resolution: 8.3 pixels mm^-1^	θ_max_ = 28.7°, θ_min_ = 2.0°
sets of exposures each taken over 0.5° ω rotation scans	*h* = −12→10
Absorption correction: multi-scan (SADABS; Sheldrick, 1996)	*k* = −13→12
*T*_min_ = 0.782, *T*_max_ = 0.915	*l* = −15→15
8767 measured reflections	

### Refinement

**Table:**

Refinement on *F*^2^	Primary atom site location: structure-invariant direct methods
Least-squares matrix: full	Secondary atom site location: difference Fourier map
*R*[*F*^2^ > 2σ(*F*^2^)] = 0.047	Hydrogen site location: inferred from neighbouring sites
*wR*(*F*^2^) = 0.159	H-atom parameters constrained
*S* = 1.04	*w* = 1/[σ^2^(*F*_o_^2^) + (0.097*P*)^2^ + 0.1562*P*] where *P* = (*F*_o_^2^ + 2*F*_c_^2^)/3
4422 reflections	(Δ/σ)_max_ = 0.002
227 parameters	Δρ_max_ = 0.44 e Å^−^^3^
0 restraints	Δρ_min_ = −0.36 e Å^−^^3^

### Special details

**Table:**

Experimental. Crystal grew over 48 h.
Geometry. All e.s.d.'s (except the e.s.d. in the dihedral angle between two l.s. planes) are estimated using the full covariance matrix. The cell e.s.d.'s are taken into account individually in the estimation of e.s.d.'s in distances, angles and torsion angles; correlations between e.s.d.'s in cell parameters are only used when they are defined by crystal symmetry. An approximate (isotropic) treatment of cell e.s.d.'s is used for estimating e.s.d.'s involving l.s. planes.
Refinement. Data were collected by measuring three sets of exposures with the detector set at 2θ = 29°, crystal-to-detector distance 6.00 cm. Refinement of *F*^2^ against ALL reflections.

### Fractional atomic coordinates and isotropic or equivalent isotropic displacement parameters (Å^2^)

**Table:**

	*x*	*y*	*z*	*U*_iso_*/*U*_eq_	
Cl1	0.64470 (8)	0.98821 (8)	0.38456 (7)	0.0691 (2)	
Cl2	0.26825 (11)	−0.01534 (7)	−0.06864 (6)	0.0787 (3)	
Cl3	0.17315 (6)	0.47020 (7)	−0.12520 (5)	0.05352 (19)	
O1	0.11480 (18)	0.6582 (2)	0.40412 (15)	0.0512 (4)	
O2	0.28993 (18)	0.62358 (19)	0.52192 (14)	0.0473 (4)	
N1	0.18786 (18)	0.67376 (17)	0.17013 (15)	0.0330 (3)	
H1	0.1142	0.6505	0.2107	0.040*	
H2	0.1838	0.5903	0.0966	0.040*	
C1	0.2347 (2)	0.6371 (2)	0.41927 (17)	0.0346 (4)	
C2	0.3435 (2)	0.6218 (2)	0.32432 (17)	0.0321 (4)	
H21	0.4452	0.6583	0.3773	0.039*	
C3	0.3372 (2)	0.7230 (2)	0.25943 (17)	0.0314 (4)	
H31	0.3497	0.8237	0.3299	0.038*	
C4	0.4634 (2)	0.7305 (2)	0.18192 (19)	0.0377 (4)	
H41	0.4571	0.6302	0.1166	0.045*	
H42	0.4464	0.7852	0.1349	0.045*	
C5	0.6210 (2)	0.8022 (3)	0.2633 (2)	0.0457 (5)	
H51	0.6930	0.8014	0.2058	0.055*	
H52	0.6418	0.7446	0.3064	0.055*	
C6	0.3211 (2)	0.4594 (2)	0.22692 (18)	0.0338 (4)	
C7	0.4464 (2)	0.4091 (3)	0.1945 (2)	0.0431 (5)	
H71	0.5425	0.4744	0.2344	0.052*	
C8	0.4308 (3)	0.2642 (3)	0.1046 (2)	0.0507 (6)	
H81	0.5154	0.2315	0.0833	0.061*	
C9	0.2888 (3)	0.1685 (2)	0.0466 (2)	0.0495 (6)	
C10	0.1623 (3)	0.2137 (3)	0.0776 (2)	0.0494 (5)	
H101	0.0668	0.1469	0.0380	0.059*	
C11	0.1778 (2)	0.3591 (2)	0.1681 (2)	0.0423 (5)	
H111	0.0925	0.3903	0.1899	0.051*	
C12	0.1968 (3)	0.6398 (3)	0.6215 (2)	0.0590 (7)	
H121	0.2542	0.6480	0.6984	0.088*	
H122	0.1100	0.5545	0.5872	0.088*	
H123	0.1646	0.7278	0.6445	0.088*	
C13	0.1553 (3)	0.7857 (2)	0.1302 (2)	0.0416 (5)	
H131	0.2240	0.7983	0.0735	0.050*	
H132	0.0535	0.7461	0.0790	0.050*	
C14	0.1697 (2)	0.9353 (2)	0.2434 (2)	0.0387 (4)	
C15	0.0533 (3)	0.9580 (3)	0.3120 (3)	0.0589 (6)	
H151	−0.0319	0.8791	0.2907	0.071*	
C16	0.0627 (4)	1.0968 (3)	0.4118 (3)	0.0726 (8)	
H161	−0.0156	1.1104	0.4583	0.087*	
C17	0.1861 (4)	1.2151 (3)	0.4434 (3)	0.0646 (7)	
H171	0.1906	1.3089	0.5097	0.077*	
C18	0.3036 (3)	1.1941 (3)	0.3761 (2)	0.0562 (6)	
H181	0.3883	1.2736	0.3974	0.067*	
C19	0.2947 (3)	1.0547 (3)	0.2771 (2)	0.0456 (5)	
H191	0.3742	1.0408	0.2322	0.055*	

### Atomic displacement parameters (Å^2^)

**Table:**

	*U*^11^	*U*^22^	*U*^33^	*U*^12^	*U*^13^	*U*^23^
Cl1	0.0507 (4)	0.0516 (4)	0.0684 (4)	−0.0046 (3)	0.0023 (3)	0.0063 (3)
Cl2	0.1402 (8)	0.0425 (4)	0.0514 (4)	0.0395 (4)	0.0239 (4)	0.0128 (3)
Cl3	0.0418 (3)	0.0546 (4)	0.0430 (3)	0.0064 (2)	0.0086 (2)	0.0077 (2)
O1	0.0410 (9)	0.0740 (12)	0.0479 (8)	0.0263 (8)	0.0165 (7)	0.0306 (8)
O2	0.0516 (9)	0.0586 (10)	0.0389 (7)	0.0191 (8)	0.0122 (6)	0.0269 (7)
N1	0.0316 (8)	0.0309 (8)	0.0339 (7)	0.0081 (6)	0.0027 (6)	0.0138 (6)
C1	0.0379 (10)	0.0281 (9)	0.0313 (9)	0.0053 (8)	0.0045 (7)	0.0103 (7)
C2	0.0278 (9)	0.0334 (10)	0.0332 (8)	0.0074 (7)	0.0039 (7)	0.0149 (7)
C3	0.0289 (9)	0.0284 (9)	0.0306 (8)	0.0048 (7)	0.0015 (7)	0.0106 (7)
C4	0.0349 (10)	0.0377 (11)	0.0375 (9)	0.0058 (8)	0.0077 (8)	0.0170 (8)
C5	0.0351 (11)	0.0457 (12)	0.0527 (12)	0.0067 (9)	0.0097 (9)	0.0220 (10)
C6	0.0390 (10)	0.0330 (10)	0.0340 (9)	0.0136 (8)	0.0097 (7)	0.0176 (8)
C7	0.0411 (11)	0.0451 (12)	0.0520 (11)	0.0188 (9)	0.0169 (9)	0.0261 (10)
C8	0.0627 (15)	0.0514 (14)	0.0564 (13)	0.0329 (12)	0.0298 (11)	0.0307 (11)
C9	0.0855 (18)	0.0354 (11)	0.0353 (10)	0.0277 (12)	0.0167 (11)	0.0175 (9)
C10	0.0565 (14)	0.0359 (11)	0.0491 (12)	0.0099 (10)	−0.0034 (10)	0.0175 (10)
C11	0.0390 (11)	0.0360 (11)	0.0491 (11)	0.0110 (9)	0.0030 (9)	0.0180 (9)
C12	0.0731 (17)	0.0678 (17)	0.0396 (11)	0.0207 (14)	0.0212 (11)	0.0263 (12)
C13	0.0464 (12)	0.0381 (11)	0.0405 (10)	0.0126 (9)	−0.0013 (8)	0.0194 (9)
C14	0.0373 (11)	0.0360 (11)	0.0447 (10)	0.0133 (8)	0.0010 (8)	0.0201 (9)
C15	0.0377 (12)	0.0492 (14)	0.0818 (17)	0.0139 (10)	0.0119 (11)	0.0227 (13)
C16	0.0671 (19)	0.0614 (18)	0.087 (2)	0.0355 (15)	0.0280 (16)	0.0221 (15)
C17	0.083 (2)	0.0421 (14)	0.0609 (15)	0.0286 (14)	0.0017 (14)	0.0136 (12)
C18	0.0662 (16)	0.0382 (12)	0.0588 (14)	0.0058 (11)	−0.0061 (12)	0.0246 (11)
C19	0.0476 (12)	0.0427 (12)	0.0502 (11)	0.0100 (9)	0.0065 (9)	0.0267 (10)

### Geometric parameters (Å, °)

**Table:**

Cl1—C5	1.776 (2)	C8—C9	1.370 (4)
Cl2—C9	1.745 (2)	C8—H81	0.9300
O1—C1	1.196 (3)	C9—C10	1.372 (3)
O2—C1	1.330 (2)	C10—C11	1.379 (3)
O2—C12	1.454 (3)	C10—H101	0.9300
N1—C3	1.502 (2)	C11—H111	0.9300
N1—C13	1.508 (3)	C12—H121	0.9600
N1—H1	0.9000	C12—H122	0.9600
N1—H2	0.9000	C12—H123	0.9600
C1—C2	1.517 (3)	C13—C14	1.501 (3)
C2—C6	1.522 (3)	C13—H131	0.9700
C2—C3	1.544 (3)	C13—H132	0.9700
C2—H21	0.9800	C14—C15	1.381 (3)
C3—C4	1.526 (3)	C14—C19	1.381 (3)
C3—H31	0.9800	C15—C16	1.376 (4)
C4—C5	1.508 (3)	C15—H151	0.9300
C4—H41	0.9700	C16—C17	1.370 (4)
C4—H42	0.9700	C16—H161	0.9300
C5—H51	0.9700	C17—C18	1.380 (4)
C5—H52	0.9700	C17—H171	0.9300
C6—C7	1.387 (3)	C18—C19	1.379 (3)
C6—C11	1.395 (3)	C18—H181	0.9300
C7—C8	1.374 (3)	C19—H191	0.9300
C7—H71	0.9300		
			
C1—O2—C12	116.08 (18)	C9—C8—H81	120.4
C3—N1—C13	115.50 (15)	C7—C8—H81	120.4
C3—N1—H1	108.4	C8—C9—C10	121.5 (2)
C13—N1—H1	108.4	C8—C9—Cl2	119.32 (19)
C3—N1—H2	108.4	C10—C9—Cl2	119.2 (2)
C13—N1—H2	108.4	C9—C10—C11	119.5 (2)
H1—N1—H2	107.5	C9—C10—H101	120.3
O1—C1—O2	124.21 (18)	C11—C10—H101	120.3
O1—C1—C2	124.68 (17)	C10—C11—C6	120.2 (2)
O2—C1—C2	111.11 (17)	C10—C11—H111	119.9
C1—C2—C6	111.18 (15)	C6—C11—H111	119.9
C1—C2—C3	111.11 (15)	O2—C12—H121	109.5
C6—C2—C3	114.25 (14)	O2—C12—H122	109.5
C1—C2—H21	106.6	H121—C12—H122	109.5
C6—C2—H21	106.6	O2—C12—H123	109.5
C3—C2—H21	106.6	H121—C12—H123	109.5
N1—C3—C4	109.14 (15)	H122—C12—H123	109.5
N1—C3—C2	111.71 (14)	C14—C13—N1	114.43 (16)
C4—C3—C2	112.34 (16)	C14—C13—H131	108.7
N1—C3—H31	107.8	N1—C13—H131	108.7
C4—C3—H31	107.8	C14—C13—H132	108.7
C2—C3—H31	107.8	N1—C13—H132	108.7
C5—C4—C3	115.28 (16)	H131—C13—H132	107.6
C5—C4—H41	108.5	C15—C14—C19	118.5 (2)
C3—C4—H41	108.5	C15—C14—C13	120.1 (2)
C5—C4—H42	108.5	C19—C14—C13	121.3 (2)
C3—C4—H42	108.5	C16—C15—C14	120.4 (2)
H41—C4—H42	107.5	C16—C15—H151	119.8
C4—C5—Cl1	111.82 (16)	C14—C15—H151	119.8
C4—C5—H51	109.3	C17—C16—C15	120.8 (3)
Cl1—C5—H51	109.3	C17—C16—H161	119.6
C4—C5—H52	109.3	C15—C16—H161	119.6
Cl1—C5—H52	109.3	C16—C17—C18	119.6 (2)
H51—C5—H52	107.9	C16—C17—H171	120.2
C7—C6—C11	118.70 (19)	C18—C17—H171	120.2
C7—C6—C2	119.31 (18)	C19—C18—C17	119.6 (2)
C11—C6—C2	121.99 (17)	C19—C18—H181	120.2
C8—C7—C6	121.0 (2)	C17—C18—H181	120.2
C8—C7—H71	119.5	C18—C19—C14	121.2 (2)
C6—C7—H71	119.5	C18—C19—H191	119.4
C9—C8—C7	119.1 (2)	C14—C19—H191	119.4
			
C1—C2—C3—C4	170.19 (15)	C3—C2—C6—C11	−84.9 (2)
C2—C3—C4—C5	−65.1 (2)	C11—C6—C7—C8	1.2 (3)
C3—C4—C5—Cl1	−59.2 (2)	C2—C6—C7—C8	−178.37 (18)
C3—C2—C1—O2	−145.64 (16)	C6—C7—C8—C9	−0.2 (3)
C2—C1—O2—C12	178.60 (18)	C7—C8—C9—C10	−0.8 (3)
C1—C2—C6—C7	−138.62 (18)	C7—C8—C9—Cl2	179.55 (17)
C2—C3—N1—C13	164.81 (16)	C8—C9—C10—C11	0.7 (3)
C3—N1—C13—C14	−53.8 (2)	Cl2—C9—C10—C11	−179.63 (16)
N1—C13—C14—C15	−80.7 (3)	C9—C10—C11—C6	0.3 (3)
C12—O2—C1—O1	−1.5 (3)	C7—C6—C11—C10	−1.3 (3)
O1—C1—C2—C6	−93.9 (2)	C2—C6—C11—C10	178.28 (18)
O2—C1—C2—C6	85.91 (19)	N1—C13—C14—C19	102.7 (2)
O1—C1—C2—C3	34.5 (3)	C19—C14—C15—C16	−0.1 (4)
C13—N1—C3—C4	−70.3 (2)	C13—C14—C15—C16	−176.8 (2)
C1—C2—C3—N1	−66.78 (18)	C14—C15—C16—C17	1.1 (5)
C6—C2—C3—N1	60.0 (2)	C15—C16—C17—C18	−1.4 (5)
C6—C2—C3—C4	−63.0 (2)	C16—C17—C18—C19	0.7 (4)
N1—C3—C4—C5	170.40 (17)	C17—C18—C19—C14	0.3 (3)
C3—C2—C6—C7	94.6 (2)	C15—C14—C19—C18	−0.6 (3)
C1—C2—C6—C11	41.8 (2)	C13—C14—C19—C18	176.10 (19)

### Hydrogen-bond geometry (Å, °)

**Table:**

*D*—H···*A*	*D*—H	H···*A*	*D*···*A*	*D*—H···*A*
N1—H2···Cl3	0.90	2.28	3.0890 (18)	150
N1—H1···Cl3^i^	0.90	2.59	3.2447 (18)	131

Symmetry codes: (i) −*x*, −*y*+1, −*z*.
